# Serum neurofilament light chain and disease-modifying treatment as predictors of cognitive decline in multiple sclerosis

**DOI:** 10.3389/fneur.2026.1743472

**Published:** 2026-02-04

**Authors:** Vera Taluntiene, Natasa Giedraitiene, Rasa Kizlaitiene, Mantas Vaisvilas, Gintaras Kaubrys

**Affiliations:** Clinic of Neurology and Neurosurgery, Institute of Clinical Medicine, Faculty of Medicine, Vilnius University, Vilnius, Lithuania

**Keywords:** 9-Hole Peg Test, BICAMS, cognitive impairment, disease-modifying treatments, multiple sclerosis, neurofilament light chain, Timed 25-Foot Walk Test

## Abstract

**Background:**

Cognitive impairment (CI) is a prevalent and early problem in multiple sclerosis (MS). Its detection and monitoring might help prevent cognitive decline and potentially inform treatment decisions.

**Objective:**

The aim of this study was to analyze the possible associations of cognitive decline and neurofilament levels, disability measures, and disease-modifying treatments (DMTs) in patients with MS (PwMS).

**Methods:**

64 PwMS and 14 healthy controls participated in this prospective study. Neurofilament light chain (NfL) levels were measured in the cerebrospinal fluid (CSF) samples of PwMS and serum samples of both groups. Serum samples, cognitive testing using Brief International Cognitive Assessment for MS (BICAMS) battery and disability assessment using EDSS, Nine-Hole Peg Test (9HPT) and Timed 25-Foot Walk Test (T25FWT) were evaluated at the time of diagnosis and at follow-up at 12 months. Longitudinal change in BICAMS subtest scores was calculated and its associations with NfL concentration, disability measures and demographic/clinical data were analyzed.

**Results:**

All PwMS were diagnosed with relapsing MS and 78.1% were started on platform DMTs, while the rest were prescribed higher-efficacy DMTs. PwMS had significantly higher sNfL concentration at baseline (11.95 vs. 5.40 pg./mL, *p* = 0.001) but follow-up sNfL levels were similar to those of control group (7.40 vs. 5.50 pg./mL, *p* = 0.768). At baseline, all BICAMS subtest scores were lower in PwMS, but the difference in the Symbol Digit Modalities Test (SDMT) was not statistically significant. However, it was the only subtest to significantly decrease at follow-up (−1.87, *p* = 0.024). Correlation analysis showed that SDMT1 and 2 were associated with serum NfL2 measurement. Logistic regression analysis demonstrated that serum NfL at baseline and follow-up were significant predictors for SDMT decline. Platform DMTs, when compared to higher-efficacy DMTs, also significantly increased the odds of SDMT decline.

**Conclusion:**

Our findings highlight the potential of serum NfL as a marker of early IPS decline. Treatment with higher-efficacy DMTs early after diagnosis may be protective against early cognitive decline in MS.

## 1 Introduction

Multiple sclerosis (MS) is a chronic autoimmune demyelinating disease of the central nervous system (CNS), commonly affecting young people. Early in the disease course, demyelination and inflammation are responsible for the clinical relapses and symptoms, while in the later stages, neurodegeneration becomes the driving force of accumulating disability (1). In addition to multifocal neurological symptoms caused by MS, cognitive impairment (CI) is frequently observed even at disease onset (2–4).

The most commonly affected cognitive domains are information processing speed (IPS), episodic memory and learning, attention, visuospatial abilities and executive functions (5). The pathogenesis of MS-related CI is likely multifactorial. The lesions of grey matter structures and normal-appearing white matter seem to play a role in CI development, as well as changes in synaptic transmission and plasticity (6). Imaging studies have reported associations of CI with the lesion load and whole brain or cortical atrophy (7). CI is an important risk factor for unemployment, withdrawal from social activities and generally worse quality of life, especially given that MS is usually diagnosed in the busiest and most productive years (8, 9).

Nowadays, the most commonly used battery for neuropsychological assessment in MS is BICAMS (Brief International Cognitive Assessment in MS) (10). BICAMS is short (up to 15 min), sensitive and easy to use in smaller centers without trained neuropsychologists. Routine cognitive evaluation is encouraged in clinical practice to employ cognitive rehabilitation techniques (11) and potentially inform treatment decisions. There is some evidence to suggest that disease-modifying treatments (DMTs) could have a protective effect on cognition in PwMS.

Early initiation of higher-efficacy DMT significantly reduces the risk of disability progression compared to delayed treatment or escalation from platform DMTs (12). Studies indicate that early use of high-efficacy DMT is associated with better long-term outcomes, including lower relapse rates and slower disease progression (12). The choice of first-line therapy is crucial for prognosis in MS patients, as suboptimal initial treatment has been linked to an increased risk of relapses, appearance of new brain lesions, and disability progression (13, 14).

Neurofilament light chain (NfL) is a subunit of a family of structural neuronal proteins, responsible for the elongation and maintenance of axonal structural integrity (15). Small NfL concentrations can be detected in the cerebrospinal fluid (CSF) and blood (serum or plasma) of healthy people, normally increasing with age (16). However, NfL concentrations rise considerably in various neurodegenerative conditions, with one being MS. NfL is associated with clinical relapses, radiological activity (new or active T2 hyperintense lesions), disability and response to DMTs in PwMS (17), therefore, it is a possible biomarker for both inflammatory activity and neurodegeneration in MS. The relationship of NfL and CI is not yet clearly established, although there are accumulating data to demonstrate the association of NfL levels and IPS impairment (18, 19).

The goal of this study was to analyze the possible associations of cognitive decline and NfL concentrations, disability measures and disease-modifying treatments in PwMS.

## 2 Materials and methods

### 2.1 Participants

This single-center, prospective longitudinal study included consecutive patients, diagnosed with MS at Vilnius University Hospital Santaros Clinics between February 2020 and August 2024, and a control group (CG) of healthy volunteers. MS was diagnosed according to the revised 2017 McDonald’s criteria. Baseline evaluation was done during remission (at least 4 weeks after relapse and/or steroid pulse therapy) and before the initiation of DMTs. We included participants between 18 and 65 years of age, who agreed to participate in the study by signing a written informed consent form. Exclusion criteria were other diagnosed CNS or psychiatric diseases producing physical disability or CI, comorbidities that might affect serum NfL levels (hypertension, diabetes, small vessel disease, other autoimmune conditions), visual or hearing impairment, history of substance abuse, unwillingness to participate in the study, technical difficulties in obtaining serum or CSF samples for analysis. CG participants were not diagnosed with MS and conformed to the same exclusion criteria as PwMS. The results of the baseline cohort are presented elsewhere, while the present study reports longitudinal findings (20). Follow-up evaluation was performed at 12 ± 1 months (depending on patients’ visit schedules). Disability, cognitive parameters and follow-up serum NfL were evaluated during remission. Data on DMTs was collected.

In Lithuania, MS treatment decisions are made according to the regulations of the Ministry of Health of Lithuania which generally follow escalation strategy. Platform medications are usually used as first-line therapy. Patients may be prescribed higher-efficacy DMTs if they experience at least one clinical relapse while on platform therapy and have either at least one gadolinium-enhancing lesion or no fewer than nine demyelinating lesions on MRI. However, a subset of patients is eligible to get higher-efficacy DMTs as first-line if they present with two or more disabling exacerbations within one year, in combination with at least one contrast-enhancing lesion or increasing number of T2 lesions on subsequent MRI. The prescription of DMTs is only limited by EDSS score – those who exceed 6 are not started on DMTs. In our study, all patients were prescribed treatment according to these regulations. Glatiramer acetate, interferons, teriflunomide and dimethyl fumarate were classified as platform DMTs, while fingolimod, ponesimod, cladribine and monoclonal antibodies – as higher-efficacy DMTs.

This study was approved by the Ethics Committee for Scientific Research of the Faculty of Medicine of Vilnius University (2020/2-1200-685; 2023-LP-91). All study participants provided informed consent in writing prior to their involvement in the research.

### 2.2 Cognitive evaluation

Cognitive abilities were tested using BICAMS (10), a set of rapid and easily repeatable tests, which consists of the Symbol Digit Modalities Test (SDMT), the Brief Visuospatial Memory Test Revised (BVMT-R) and the California Verbal Learning Test II (CVLT-II).

SDMT evaluates IPS (21). The test was performed on a worksheet with the key at the top, where nine symbols were paired with numbers. The rest of the worksheet consisted of pseudo-randomly arranged sequence of those symbols. Initially, participants analyzed the symbol-number pairings in the key, then they dictated as many corresponding numbers as possible for 90 s.

BVMT-R is a test of immediate visual recall and learning (22). Participants were presented with six abstract geometric figures on the sheet of paper, arranged in three rows of two for ten seconds. Then they were required to reproduce the figures on a different sheet of paper. Points were allocated for the correct shape (one point) and location (one point) of every figure. The final result was the sum of three trials (maximum score being 36).

Immediate verbal recall and learning was tested using CVLT-II (23), where the investigator read a randomly arranged 16-word list, consisting of four semantic categories with four items in each category. Participants were required to recall as many items as possible after each of five readings. The final score was the total amount of items recalled during all trials.

Alternate versions of all BICAMS tests were used at follow-up to mitigate learning effect. CG results were used to calculate Z-scores of PwMS using medians/means and interquartile range [IQR]/standard deviation (SD). Impairment on each BICAMS test was defined as a Z-score below −1.5 SD. Overall cognitive impairment was defined as at least one abnormal cognitive score out of three BICAMS tests.

### 2.3 Disability evaluation

Physical disability was assessed using EDSS (24) scores at baseline and follow-up. Ambulation and lower extremity function was evaluated using Timed 25-Foot Walk Test (T25FWT) (25). This test records the mean time needed to safely walk 25 feet during two trials. Manual dexterity was evaluated using 9-Hole Peg Test (9HPT) (26). Testing board has a hollow containing nine pegs on one side, and nine holes on the other side. Participants were required to rapidly arrange pegs into holes and then bring them back to the hollow twice with each hand. The mean time of all trials was recorded. Shorter time to complete both T25FWT and 9HPT meant better ambulation and manual dexterity.

### 2.4 Laboratory testing

CSF NfL concentration was analyzed using ELISA immunoassay (NF-light®, UmanDiagnostics, Umea, Sweden; distributed by IBL International GMBH, Germany).

Serum samples were analyzed using Simoa assay (NFlight by Quanterix).

### 2.5 Statistical analysis

Mean ± standard deviation or median [IQR] were used to present the data. Normality of distributions was assessed by Shapiro–Wilk test. For nominal variables, group differences were calculated using *Chi* square or Fisher’s exact test, while string variables were compared using Student’s *T*-test or Mann–Whitney U test, depending on the distribution. Longitudinal measurements were compared using related samples *T*-test for normally distributed variables and related samples Wilcoxon signed rank test for non-normally distributed variables. Age-adjusted Z-scores were calculated for cognitive and motor test scores and neurofilament concentration (except for CSF NfL, where age adjustment using CG results was not available). Non-normally distributed variables were further analyzed after log-transformation.

IBM SPSS Statistics v. 30 was used for all statistical analyses. The significance threshold was set at *p* < 0.05.

## 3 Results

### 3.1 Clinical and laboratory outcomes

64 PwMS and 14 CG participated in the study. The groups did not differ in demographic characteristics such as age (PwMS min. 18, max. 61; CG min. 26, max. 61 years old), education or gender ratio (Table 1). Table 1 also includes variables previously reported to influence serum NfL levels such as relapse phenotype or topography and contrast enhancement.

**Table 1:** Demographic, clinical, and laboratory characteristics of study participants.

Variable	MS (*n* = 64)^1^	CG (*n* = 14)^1^	*p*
Female	42 (65.62)	8 (57.14)	0.549
Age, years	33.50 [20.00]	33.00 [20.00]	0.768
Education, years	16.00 [3.50]	18.00 [5.00]	0.078
Disease severity/disability			
EDSS1	2.50 [1.50]	-	-
EDSS2	3.00 [2.00]	-	
EDSS change, *p*	**0.002**	-	**-**
9HPT1, sec	22.81 [5.65]	18.58 [1.79]	**0.001**
9HPT2, sec	22.11 [5.42]	17.35 [3.34]	**0.015**
9HPT change, *p*	0.164	0.279	-
T25FWT1, sec	4.13 [1.52]	3.47 [1.34]	0.124
T25FWT2, sec	4.22 [1.55]	3.49 [1.23]	**0.037**
T25FWT change, *p*	0.098	0.533	-
Phenotype/topography of previous relapse			
Motor involvement	51 (79.69)	-	-
Spinal involvement	14 (21.88)	-	-
Brainstem involvement	11 (17.19)	-	-
Contrast-enhancement on MRI	25 (39.06)	-	-
DMTs			
Platform	50 (78.12)		
Higher efficacy	14 (21.88)	-	-
DMT switch during follow-up period	24 (48.00)		
Due to relapses	11 (45.83)		
Due to AE or intolerance	13 (54.17)	-	-
CSF NfL, ng/mL	938.33 [1435.00]	-	-
sNfL1, pg./mL	11.95 [14.20]	5.40 [3.90]	**0.001**
sNfL2, pg./mL	7.40 [5.30] ***p* < 0.001**	5.50 [5.50] *p* = 0.071	0.768

^1^Median [IQR]; Mean ± SD; *n* (%).

AE, adverse events; CG, control group; CSF, cerebrospinal fluid; DMTs, disease-modifying treatments; EDSS, Expanded Disability Status Scale; IQR, interquartile range; MRI, magnetic resonance imaging; MS, multiple sclerosis; ng/mL, nanograms per milliliter; NfL, neurofilament light chain; pg/mL, picograms per milliliter; s, serum; sec, seconds; RMS, relapsing MS; T25FWT, Timed 25-Foot Walk Test; 9HPT, 9-Hole Peg Test.

Significant *p* values (<0.05) in bold.

1 and 2 represent baseline and follow-up evaluations.

All patients were diagnosed with RMS. After diagnosis, 78.12% of PwMS were started on platform DMTs and 21.88% were given higher-efficacy DMTs. Almost half of PwMS on platform DMTs switched their medication during the follow-up period: 45.8% due to relapses, while the others – because of intolerance or DMT-related adverse events (AEs). Those who experienced relapses on platform DMTs were escalated to higher-efficacy DMTs, in accordance to the national guidelines. Median EDSS increased from 2.5 to 3.0 during the follow-up period, and the difference was significant (*p* = 0.002).

Since some PwMS were initiated on higher-efficacy DMTs according to the national guidelines described above, we compared these patients with those receiving platform therapies with respect to baseline demographic, clinical, laboratory, and radiological characteristics (Table 2). The only statistically significant difference between the groups was baseline 9HPT performance, which was worse in the higher-efficacy DMT group (26.74 [5.85] vs. 22.46 [5.26] sec). The difference in T25FWT scores also approached statistical significance (4.70 [1.42] vs. 3.96 [1.39] sec, *p* = 0.067). Given these baseline differences in motor performance, all subsequent regression analyses were adjusted for motor disability by including one of the following covariates: 9HPT, T25FWT, or EDSS.

**Table 2:** Comparison of patients from platform vs. higher-efficacy DMT groups.

Variable	Platform (*n* = 50)^1^	Higher-efficacy (*n* = 14)^1^	*p*
Age, years	31.50 [19.00]	38.00 [15.50]	0.435
EDSS1	2.00 [1.38]	3.00 [2.00]	0.181
EDSS at previous relapse	3.00 [1.00]	3.00 [2.00]	0.461
9HPT1, sec	22.46 [5.26]	26.74 [5.85]	**0.014**
T25FWT1, sec	3.96 [1.39]	4.70 [1.42]	*0.067*
CSF NfL, ng/mL	942.06 [1263.73]	661.86 [1797.10]	0.445
sNfL1, pg./mL	11.95 [13.62]	12.30 [13.80]	0.826
sNfL2, pg./mL	7.35 [4.88]	7.90 [4.60]	0.475
BVMT1	29.00 [8.0]	26.00 [10.3]	0.275
SDMT1	49.74 ± 11.69	46.36 ± 8.98	0.256
CVLT1	54.77 ± 10.52	50.07 ± 9.23	0.118
Motor involvement	40 (80.00)	11 (78.57)	>0.999
Spinal involvement	10 (20.00)	4 (28.57)	0.765
Brainstem involvement	8 (16.00)	3 (21.43)	0.485
Contrast-enhancing lesions	19 (38.00)	6 (42.86)	0.765

^1^Median [IQR]; Mean ± SD; *n* (%).

BVMT, Brief Visuospatial Memory Test – Revised; CSF, cerebrospinal fluid; CVLT, California Verbal Learning Test II edition; DMTs, disease-modifying treatments; EDSS, Expanded Disability Status Scale; IQR, interquartile range; ng/mL, nanograms per mililiter; NfL, neurofilament light chain; pg/mL, picograms per mililiter; s, serum; sec, seconds; SD, standard deviation; SDMT, Symbol Digit Modalities Test; T25FWT, Timed 25-Foot Walk test; 9HPT, 9-Hole Peg Test.

Significant *p* values (<0.05) in bold.

Baseline median sNfL concentration was significantly higher in PwMS (11.95 vs. 5.40 pg./mL). Follow-up sNfL concentrations in this group decreased markedly, losing the significant difference from the CG (7.40 vs. 5.50 pg./mL). The difference between sNfL1 and sNfL2 concentrations of PwMS was statistically significant (*p* < 0.001), while CG concentrations remained stable. Median CSF NfL concentration was 938.33 ng/mL. A moderate positive correlation was found between CSF NfL and sNfL at baseline (Spearman’s *rho* 0.621, *p* < 0.001).

Comparing the motor scores of PwMS and CG, 9HPT completion times were significantly longer in PwMS both at baseline and follow-up (*p* = 0.001 and *p* = 0.015, respectively), demonstrating worse hand dexterity. Ambulation scores were worse in PwMS at baseline, although the difference was not significant at that timepoint (4.13 vs. 3.47 s, *p* = 0.124). However, at follow-up, scores of PwMS worsened (a trend toward statistical significance, *p* = 0.098), resulting in the significantly higher scores compared to CG (4.22 vs. 3.49 s, *p* = 0.037).

### 3.2 Cognitive outcomes

SDMT1 scores were higher in CG (54.64 vs. 49.00) but there was only a trend toward statistical significance (*p* = 0.089) (Table 3). At follow-up, the scores of PwMS decreased significantly (SDMT change −1.87 ± 6.35, *p* = 0.024), while CG showed a trend toward performing better the second time (+4.00 ± 8.28, *p* = 0.069). Consequently, SDMT2 scores were significantly higher in CG. 15.60% of PwMS at baseline and 31.30% at follow-up had abnormal SDMT results.

**Table 3:** Results of cognitive testing.

Test	PwMS	CG	*p*
SDMT1, mean ± SD	49.35 ± 10.96	54.64 ± 10.58	0.089
SDMT2, mean ± SD	47.48 ± 10.84	58.64 ± 9.22	**<0.001**
SDMT change, mean ± SD	−1.87 ± 6.35	+4.00 ± 8.28	PwMS: **0.024** CG: 0.069
BVMT1, median [IQR]	27.50 [9.00]	32.00 [5.00]	**0.025**
BVMT2, median [IQR]	30.00 [6.00]	30.00 [7.00]	0.978
BVMT change, median [IQR]	+1.00 [7.50]	−2.50 [5.50]	PwMS: 0.189 CG: **0.017**
CVLT1, mean ± SD	53.52 ± 10.38	64.14 ± 7.92	**<0.001**
CVLT2, mean ± SD	54.48 ± 10.36	67.71 ± 8.04	**<0.001**
CVLT change, mean ± SD	+0.95 ± 8.31	+3.57 ± 6.74	PwMS: 0.366 CG: 0.069
CI1, *n* (%)	33 (51.60)	2 (14.30)	**0.011**
CI2, *n* (%)	42 (65.60)	4 (28.60)	**0.011**

BICAMS, Brief International Cognitive Assessment for Multiple Sclerosis; BVMT, Brief Visuospatial Memory Test – Revised; CG, control group; CI, overall cognitive impairment; CVLT, California Verbal Learning Test II edition; IQR, interquartile range; PwMS, patients with multiple sclerosis; SD, standard deviation.

Significant *p* values (<0.05) in bold.

1 and 2 represent baseline and follow-up evaluations.

Regarding BVMT scores, they were significantly higher in CG at baseline (28.00 vs. 32.00, *p* = 0.025). The second evaluation produced identical median scores in both groups (30.00, *p* = 0.978). This effect was mostly due to CG score reduction at follow-up (*p* = 0.017). 23.4% of PwMS were below −1.5 SD at baseline, and 20.30% - at follow-up.

CVLT scores of PwMS were significantly lower at both evaluations (*p* < 0.001). The longitudinal change in both groups was positive (CVLT scores were better at the second timepoint), but no significant difference was found in either group. 39.1% of PwMS had abnormal CVLT1 scores and 53.1% - abnormal CVLT2 scores.

Overall CI was detected in 51.6% of PwMS at baseline and 65.6% at follow-up. We did not find a statistically significant difference in the concentration of sNfL1, sNfL2 or CSF NfL between CI and non-CI groups (regarding overall cognition and each test individually).

### 3.3 Correlations and regression analyses

For further analysis, Z-scores of log-transformed NfL concentrations and motor tests were used. The correlations of BICAMS scores, demographic/disability parameters and NfL concentrations are presented in Table 4. CSF NfL concentrations did not correlate with any of the 3 cognitive tests. Serum NfL2 showed a moderate negative correlation with SDMT1 and 2. Similarly, both motor tests and EDSS1 and EDSS2 had moderate-to-strong negative correlations with SDMT1 and SDMT2. Older age was associated with worse cognitive scores in all the tests, while higher education correlated with better BVMT1, BVMT2 and CVLT2 scores. EDSS1, EDSS2 and sNfL1 showed a moderate negative correlation with SDMT1 and SDMT2.

**Table 4:** Correlation coefficients of BICAMS scores and demographic, laboratory, and disability measurements in PwMS.

Variables	SDMT1	SDMT2	BVMT1	BVMT2	CVLT1	CVLT2
Age	**−0.534**	**−0.519**	−0.043	**−0.268**	**−0.301**	−0.194
Education	0.241	0.203	**0.351**	**0.306**	0.131	**0.347**
EDSS1	**−0.347**	**−0.332**	0.221	−0.172	−0.114	0.093
EDSS2	**−0.438**	**−0.421**	0.099	−0.136	−0.053	0.000
CSF NfL	0.179	0.122	0.117	0.042	**0.307**	0.166
sNfL1	−0.098	−0.173	0.046	0.051	0.084	0.045
sNfL2	**−0.373**	**−0.399**	0.224	0.007	−0.076	0.055
9HPT1	**−0.512**	**−0.499**	−0.100	−0.195	**−0.335**	−0.161
9HPT2	**−0.615**	**−0.543**	−0.118	−0.204	−0.211	**−**0.152
T25FWT1	**−0.534**	**−0.481**	−0.203	**−0.381**	**−0.365**	**−0.320**
T25FWT2	**−0.400**	**−0.376**	−0.126	**−0.293**	−0.255	**−0.308**

BVMT, Brief Visuospatial Memory Test – Revised; CSF, cerebrospinal fluid; CVLT, California Verbal Learning Test II edition; EDSS, Expanded Disability Status Scale; SDMT, Symbol Digit Modalities Test; sNfL, serum neurofilament light chain; T25FWT, Timed 25-Foot Walk test; 9HPT, 9-Hole Peg Test.

*p*, Spearman’s rho. Significant correlation coefficients in bold.

PwMS were divided into groups according to the longitudinal cognitive testing changes – SDMT/CVLT/BVMT decline or stability. Mean concentrations of CSF and serum NfL of those groups are shown in Table 5. PwMS who showed a longitudinal decline in SDMT scores had higher log-transformed sNfL2 Z-scores compared to those, whose SDMT remained stable or improved (a trend toward significance, *p* = 0.059). No other significant differences were found between the groups.

**Table 5:** Groups according to changes in cognitive test scores.

Test	SDMT			CVLT			BVMT			Overall cognition		
Change	↓	=	*p*	↓	=	*p*	↓	=	*p*	↓	=	*p*
sNfL1, mean ± SD	0.31 ± 0.95	0.10 ± 1.06	0.412	0.09 ± 1.05	0.25 ± 0.91	0.540	0.42 ± 1.00	0.08 ± 1.00	0.216	0.23 ± 1.03	0.10 ± 0.76	0.636
sNfL2, mean ± SD	0.28 ± 1.06	−0.25 ± 1.12	**0.059**	−0.08 ± 1.02	0.14 ± 1.13	0.409	−0.04 ± 1.15	0.01 ± 1.06	0.886	0.10 ± 1.07	−0.21 ± 0.94	0.375
CSF NfL, mean ± SD	6.94 ± 0.96	6.88 ± 0.84	0.816	6.82 ± 0.99	6.94 ± 0.89	0.630	6.99 ± 0.88	6.78 ± 0.88	0.414	6.88 ± 0.94	6.89 ± 0.86	0.981

BVMT, Brief Visuospatial Memory Test – Revised; CSF, cerebrospinal fluid; CVLT, California Verbal Learning Test II edition; SD, standard deviation; SDMT, Symbol Digit Modalities Test; sNfL, serum neurofilament light chain.

*p*, Student’s *t*-test, significant < 0.05 in bold.

Since only SDMT decline was associated with NfL concentrations, logistic regression analysis was used to determine which independent variables significantly predicted SDMT longitudinal decrease. Various models were constructed using demographic parameters, disability/motor scores, serum/CSF NfL scores and DMT type (platform/higher-efficacy). Sex and education were included in every model, while age was only included in the models with CSF NfL, since these were not age-adjusted due to the lack of CG scores.

Two models were found to be statistically significant (Table 6). First significant model, besides demographic and therapeutic variables, included sNfL1 and T25FWT2 scores [χ^2^(5) = 14.51, *p* = 0.013]. It explained 31.5% of the variance in SDMT decline (Nagelkerke R^2^ = 0.315) and correctly classified 68.5% of cases. Higher log-transformed sNfL1 Z-scores were a significant predictor of SDMT decline (OR 2.77, 95% CI [1.25–6.15], *p* = 0.012). Among covariates, platform DMTs were also a significant predictor of SDMT decline (OR 8.52, 95% CI [1.44–50.26], *p* = 0.018), while education, sex and T25FWT2 scores were not.

**Table 6:** Multivariable logistic regression models for SDMT decline.

Characteristic	Model 1			Model 2		
	OR	95% CI	*p*	OR	95% CI	*p*
Sex	0.85	0.22, 3.09	0.807	1.28	0.35, 4.61	0.705
Education	1.25	0.98, 1.64	0.082	1.16	0.91, 1.49	0.243
DMT category: Platform therapy	8.52	1.44, 50.26	**0.018**	2.26	0.54, 10.0	0.267
sNfL1	2.77	1.25, 6.15	**0.012**	-	-	**-**
T25FWT2	2.04	0.25, 20.6	0.515	-	-	**-**
sNfL2	-	-	-	1.93	1.03, 3.61	**0.040**
T25FWT1	-	-	-	0.99	0.09, 11.0	0.992

CI, confidence interval; DMT, disease modifying treatment; NfL, neurofilament light chain; OR, Odd’s ratio; s, serum, SDMT, Symbol Digit Modalities Test; T25FWT, Timed 25-Foot Walk Test.

Significant *p* values (<0.05) in bold.

Another model, which included sNfL2 and T25FWT1, was also significant [χ^2^(4) = 9.88, *p* = 0.042]. It explained 22.8% of the variance in SDMT decline (Nagelkerke R^2^ = 0.228) and correctly classified 64.2% of cases. Higher log-transformed sNfL2 Z-scores were significantly associated with the increased odds of SDMT decline (OR 1.93, 95% CI [1.03–3.61, *p* = 0.040]). No other covariates were significant predictors of SDMT decline.

One standard deviation increase in log-transformed sNfL1 score corresponded to 2.66-fold increase in raw sNfL1 concentration. Therefore, participants with 2.66 times higher baseline sNfL1 concentration had almost three-times (2.77) higher odds of SDMT decline. Similarly, 1 standard deviation increase in log-transformed sNfL2 score corresponded to 2.9-fold difference in raw sNfL2 levels. This finding suggests that approximately a threefold increase in sNfL concentration at follow-up nearly doubled the odds of longitudinal SDMT decline.

## 4 Discussion

This prospective single-center study aimed to analyze the association between longitudinal cognitive measures, serum NfL concentration dynamics and disability measures in PwMS. In this longitudinal cohort, we confirmed that serum NfL levels are associated with IPS decline, as measured by SDMT.

The median CSF NfL concentration was close to the mean values for MS reported in a meta-analysis (27). It was almost twice the upper normal range for individuals around age 30 and roughly corresponded to the upper normal level of individuals aged 50 to 60 years (28).

Serum NfL concentration at baseline was significantly higher in PwMS compared to the CG and had moderate correlation with CSF NfL, in line with other studies (29, 30). However, follow-up sNfL concentration decreased substantially, reaching normal levels, characteristic to the CG. We hypothesize that age and MS-related sNfL increase was mitigated by the lowering effect of DMTs to which all PwMS were subjected after diagnosis, consistent with previously described data (29, 30).

NfL levels are influenced by disease severity and acute inflammatory activity, including gadolinium-enhancing lesions and relapse phenotype. Although we did not specifically investigate determinants of serum NfL in this study, we sought to increase transparency by presenting baseline inflammatory and clinical characteristics in Table 1. To mitigate potential confounding by disease severity, multivariable models of cognitive outcomes included EDSS scores or measures of motor performance, which likely capture a substantial proportion of inflammation-related disease burden.

9HPT scores at both timepoints differentiated PwMS from controls, while T25FWT scores were similar at baseline but significantly worse in PwMS at follow-up. The fact that both tests were significantly worse in PwMS at follow-up is consistent with their significant longitudinal EDSS increase. The finding that subtle manual dexterity changes, as measured by 9HPT, may be detected earlier, while ambulation may yet be normal, is in line with published data (31, 32).

PwMS got lower scores on all BICAMS tests at baseline, but the difference of SDMT1 scores was not significant. Nonetheless, out of three tests, only SDMT showed longitudinal decrease, resulting in better differentiation between the groups at follow-up. Mean CVLT-II scores increased in both groups, suggesting practice effect. Although participants were given alternate word lists at longitudinal testing, they might have remembered the general principle of the test to present four different word categories. Still, CVLT-II scores at both timepoints differentiated PwMS from CG well. BVMT-R scores were worse in PwMS at baseline, but identical at follow-up.

Although PwMS tend to get worse scores on all BICAMS subtests, SDMT is generally considered to be the most sensitive measure of cognitive dysfunction (2, 33), since IPS impairment is the most commonly affected cognitive domain (2). It has been demonstrated in newly diagnosed patients (34) and even in cases of clinically or radiologically isolated syndromes [35.6% of CIS patients in one study (35), 20–25% of CIS and RIS patients in another (36)]. In our cohort, the proportion of IPS impairment at baseline was 15.60%, which is slightly lower than in the aforementioned studies.

A previously published study of PwMS in Lithuanian population reported lower BICAMS scores in all subtests, compared to the CG. Notably, that study included older patients with mean disease duration of 11.7 years and mean EDSS score of 3.3 (37). It can be hypothesized that our PwMS had reasonably low proportion of IPS impairment at baseline due to young age, relatively high education levels and short time after diagnosis. In about a year, the rates of IPS impairment doubled. This fact highlights the importance of early interventions because this short period of time after diagnosis could be critical for preventing future cognitive decline.

Overall CI levels were relatively high in our population (51.60% at baseline and 65.60% at follow-up). This is on the higher end of spectrum for newly diagnosed PwMS but not unusually high. Up to 65% of patients with MS have been reported to have CI at baseline (38), although a pooled prevalence of CI in RMS was recently estimated to be about 32.5% (39). Wide amplitudes of reported CI in MS might stem from different MS populations (CIS, RMS, secondary progressive MS (SPMS)) at different stages of the disease and different CI evaluation methodologies.

Correlation analysis showed that lower SDMT scores at both timepoints were associated with higher disability and worse motor scores. It was also the only cognitive test associated with sNfL2. Further regression analysis demonstrated that sNfL measurements at both timepoints significantly predicted longitudinal SDMT decline. We hypothesize that higher baseline sNfL might reflect more intense inflammatory activity in the CNS at the time of diagnosis, which provokes faster neurodegeneration and brain atrophy. This manifests as IPS impairment. Although follow-up sNfL decrease could have been produced by the DMTs, the relationship with SDMT decline was still present and significant. It can be theorized that follow-up sNfL might better reflect true scale of neurodegeneration, with inflammatory effect removed.

The number of published papers with longitudinal sNfL and BICAMS assessment is limited. Jakimovski et al. reported correlation of sNfL with current and future IPS impairment, while Chitnis et al. and Friedova et al. did not find the association of sNfL and longitudinal cognitive decline (40, 41). On the other hand, EXPAND study of Siponimod vs. placebo in SPMS found that baseline sNfL was associated with lower SDMT scores at baseline and 41% increased risk of reaching 6-month confirmed worsening on SDMT (defined as 4 points from baseline) (42). In our study, the mean SDMT decline was smaller (−1.87 points), but the difference was still statistically significant. Overall, the data on the association of sNfL and cognition is mixed. Although there might be some association of sNfL and cognitive parameters, especially SDMT, sNfL is unlikely to be a singular specific marker of CI in MS (43). It might therefore be useful to include sNfL in combination with other molecular, MRI or clinical markers.

Another significant predictor of SDMT decline in logistic regression analysis was platform DMT group, which increased the odds of cognitive decline up to eight times, compared with higher-efficacy treatments. Adjustment for baseline motor impairment helped to mitigate confounding by indication related to treatment selection. This important finding is in line with current knowledge that higher- efficacy or escalation DMTs might have protective effect on cognition not only by reducing inflammation but also by the prevention or normalization of brain atrophy (44, 45).

One of limitations of our study was the small sample size, which reduced the statistical power of the statistical analyses. Moreover, a 12-month follow-up period is relatively short for a chronic disease such as multiple sclerosis. This duration may be insufficient to capture sustained cognitive changes, thereby limiting the strength of our conclusions regarding the association between cognition and treatment. Another limitation is that a lot of patients in our study switched medications during follow-up period. Such dynamic exposure to different DMTs is difficult to account for in statistical analysis. Additionally, broad categorization into platform vs. higher-efficacy DMTs does not take into consideration heterogeneous mechanisms of action, which in turn may confound the observed associations. Residual confounding by acute inflammatory features influencing serum NfL levels cannot be fully excluded. Our study also did not take into account more radiological data or depression, fatigue and anxiety evaluations which are known to affect cognitive performance (46, 47).

In conclusion, higher serum NfL might be associated with IPS impairment, seen as SDMT decline over 12 months period. Our findings suggest that initiating treatment with higher-efficacy DMTs early after diagnosis may protect against first year cognitive decline, supporting the growing body of evidence advocating for an ‘early intensive therapy’ approach in MS.

## Data Availability

The raw data supporting the conclusions of this article will be made available by the authors, without undue reservation.
